# Supplementary material for: Insulin post-transcriptionally modulates Bmal1 protein to affect the hepatic circadian clock
Source: Nat Commun. 2016 Aug 31;7:12696. doi: 10.1038/ncomms12696 (PMC5013695; doi:10.1038/ncomms12696)
Supplement: Supplementary Information — Supplementary Figures 1-5 and Supplementary Tables 1-3 [file ncomms12696-s1.pdf]

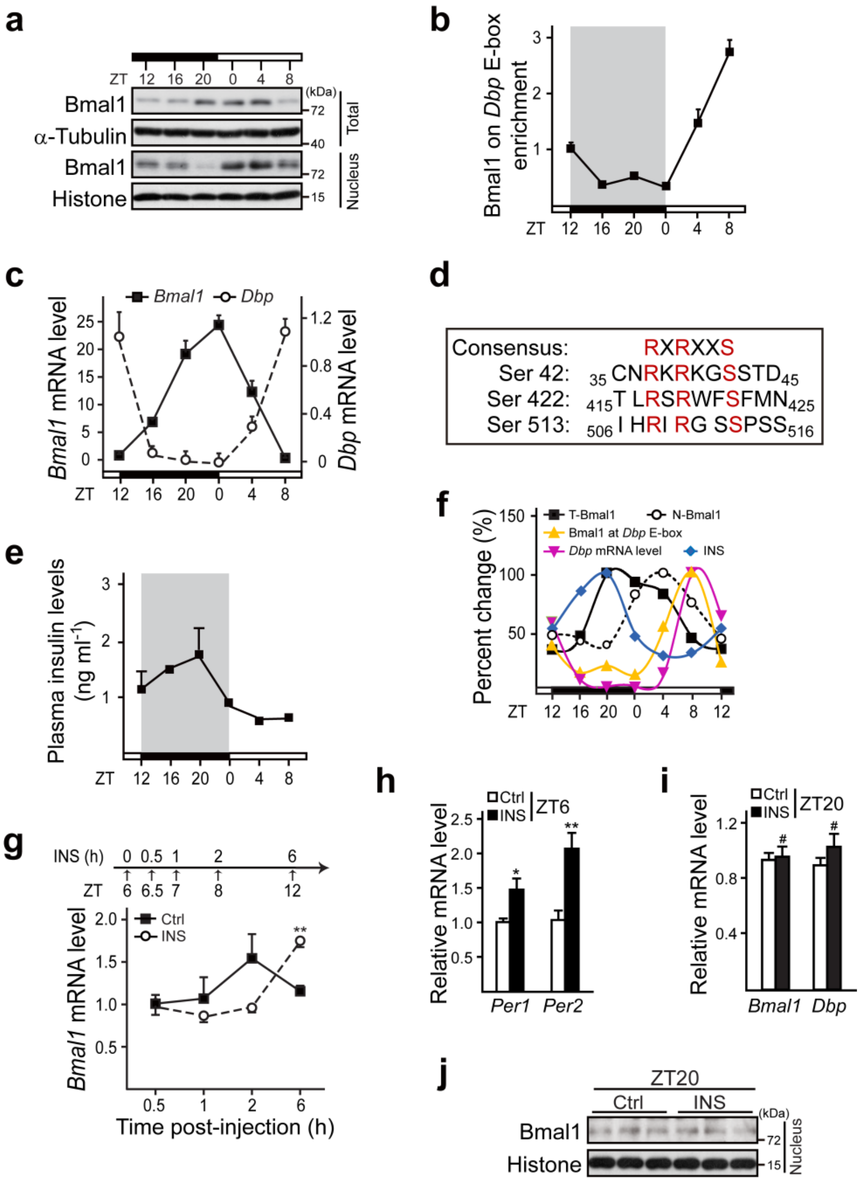

Supplementary Figure 1. Insulin affects hepatic Bmal1 nuclear accumulation

Ad libitum-fed mice were euthanized at 4 hour intervals. Liver and plasma samples were collected and prepared (a-c, e, f; n=3).

(a) Immunoblot showing total and nuclear Bmal1 protein amounts exhibit strikingly different circadian profile.

(b) ChIP analysis of the occupancy of Bmal1 on *Dbp* promoter throughout the circadian cycle.

(c) Quantitative PCR analysis of hepatic mRNA levels of *Bmal1* and *Dbp*.

(d) Schematic of relative positions of the three putative AKT consensus sites in Bmal1. The consensus recognition motifs for AKT were indicated.

(e) Measurement of plasma insulin levels.

(f) Schematic summary of the densitometries of total (black square) and nuclear (blank circle) Bmal1 protein levels, plasma insulin levels (blue diamond), Bmal1 accumulations on *Dbp* promoter (saffron yellow triangle) and *Dbp* mRNA levels (purple red triangle), which corresponds to the above results.

(g) Experimental schedule (top) of insulin injection experiments shown in Fig. 1d-f. Mice were

fasted from ZT0, injected intraperitoneally with insulin (INS, 2 U kg^{-1}) at ZT6 and then euthanized at time points indicated with small arrows (n=3). Quantitative PCR analysis of *Bmal1* mRNA levels (bottom).

(h) Quantitative PCR analysis of hepatic *Per1* and *Per2* mRNA levels. Mice were fasted from ZT0 and injected intraperitoneally with insulin (2 U kg^{-1}) or normal saline at ZT4, then animals were euthanized at ZT6 (n=4).

Ad libitum-fed mice were injected intraperitoneally with insulin (INS, 2 U kg^{-1}) at ZT18 and then euthanized at ZT20 (i-j, n=3).

(i) Quantitative PCR analysis of *Bmal1* and *Dbp* mRNA levels;

(j) Immunoblotting analysis of Bmal1 protein abundance in liver nuclear extracts. Data are represented as mean ± s.e.m, statistical analyses were performed with a two-tailed unpaired Student’s *t* test, * P<0.05, ** P<0.01, # no significant difference.

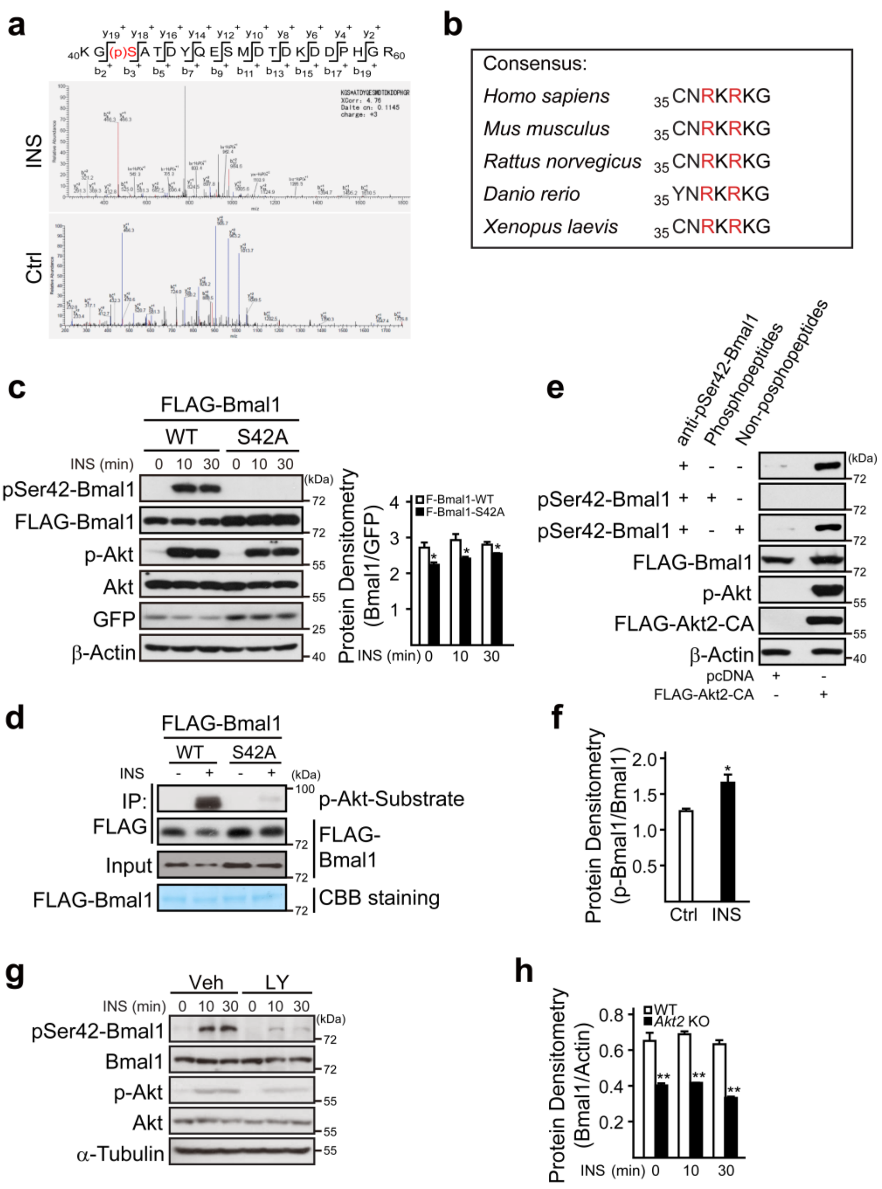

Supplementary Figure 2. Insulin induces Bmal1 Ser42 phosphorylation

(a) Mass spectrometry analysis of the phosphorylation site(s) on Bmal1. The peptide containing Ser42 was recovered from immunoprecipitates of FLAG-Bmal1 from primary hepatocytes exposed to insulin (INS, 50 nM) for 0 (Ctrl) and 30 (INS) min.

(b) Sequence alignment of *homo sapiens*, *mus musculus*, *rattus norvegicus*, *danio rerio* and *xenopus laevis* Bmal1 Ser42 with AKT consensus motif highlighted.

(c) Immunoblotting analysis of Ser42-phosphorylated Bmal1 (pSer42-Bmal1) protein amounts detected by phospho (Ser42) specific Bmal1 antiserum in Ad-FLAG-tagged wild-type Bmal1 (WT) or mutant Ser42Ala Bmal1 (S42A) virus-infected primary hepatocytes in the presence or absence of insulin (INS, 50 nM, 30 min, left), and corresponding densitometry analysis of relative Bmal1 protein amounts shown on the right (n=3)..

(d) Immunoblotting analysis of proteins recognized by phospho-Ser/Thr-Akt substrate antiserum in FLAG immunoprecipitates (IP) from lysates of primary hepatocytes infected with Ad-FLAG-tagged wild-type Bmal1 or Bmal1 (Ser42Ala) in the presence or absence of insulin (INS, 50 nM, 30min).

(e) Immunoblotting analysis of pSer42-Bmal1 protein levels in lysates of HEK 293T cells transfected with FLAG-Bmal1 together with pcDNA-FLAG or FLAG-Akt2-CA as indicated. Whole cell lysates were prepared and probed with the anti-pSer42-Bmal1 antiserum in the presence of phospho- (RKRKG(pS)ATDYQE) or non-phosphopeptides (RKRKGSATDYQE).
(f) Densitometry analysis of relative Bmal1 protein amounts corresponding to immunoblotting results of Fig. 2e (n=3).
(g) Immunoblotting analysis of Ser42-phosphorylated Bmal1 (pSer42-Bmal1) protein amounts detected by phospho (Ser42) specific Bmal1 antiserum in Ad-FLAG-WT Bmal1 or mutant S42A Bmal1 virus-infected primary hepatocytes pretreated with PI3K inhibitor LY294002 (LY, 10 nM, 2h) and followed by insulin treatment (INS, 50 nM, 30min).
(h) Densitometry analysis of relative Bmal1 protein amounts corresponding to immunoblotting results of Fig. 2f (n=3). Data are represented as mean ± s.e.m, statistical analyses were performed with a two-tailed unpaired Student's *t* test, * $P<0.05$, ** $P<0.01$.

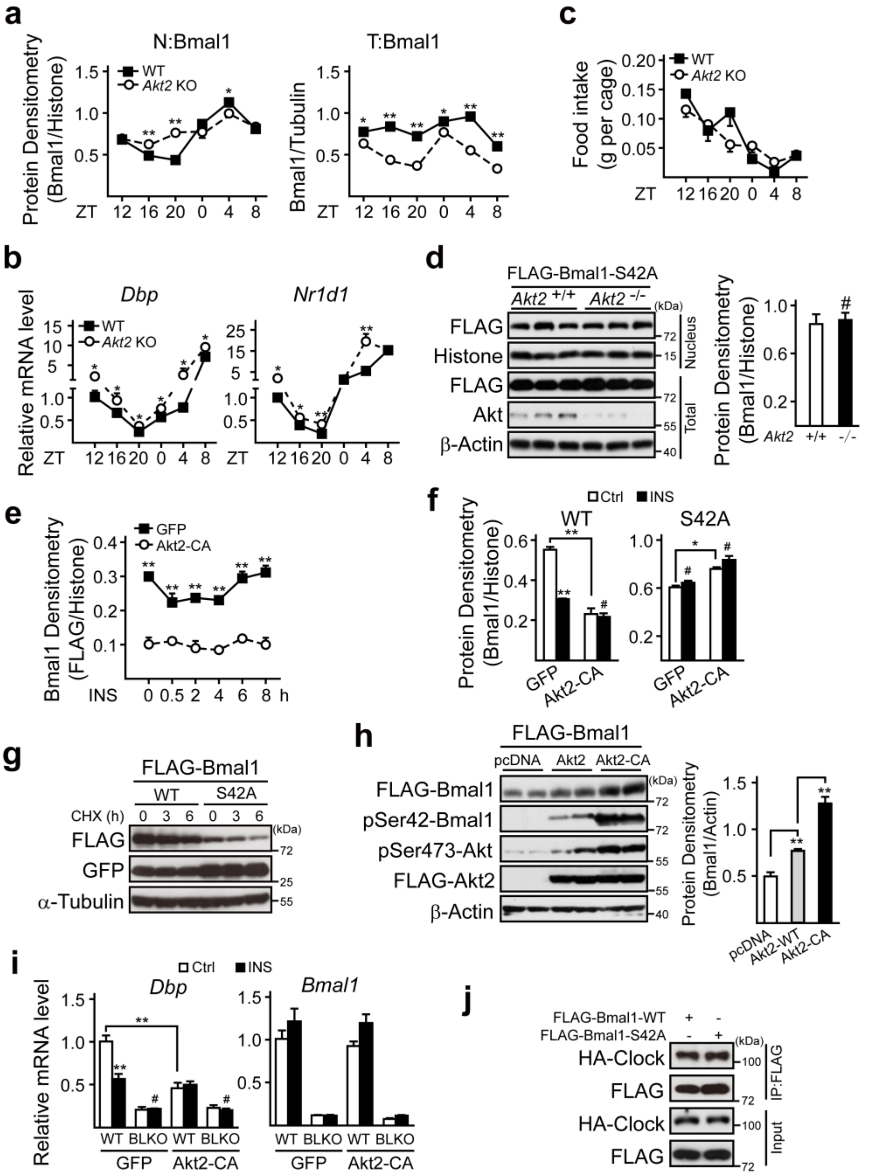

Supplementary Figure 3. The effect of Ser42 phosphorylation on Bmal1

(a) Densitometry analysis of relative nuclear Bmal1 protein amounts corresponding to immunoblotting results of Fig. 3a (n=3).

(b) Quantitative PCR analysis of hepatic mRNA levels of *Dbp* and *Nr1d1* in ad libitum-fed WT or *Akt2* KO mice euthanized at 4 hr intervals around the clock (n=3).

(c) Measurement of food intake circadian rhythm of ad libitum-fed WT or *Akt2* KO mice (3 mice per cage, n=12).

(d) Immunoblotting analysis of nuclear FLAG-Bmal1-S42A protein abundance in livers from WT (*Akt2* $^{+/+}$) or *Akt2* KO (*Akt2* $^{-/-}$) mice under ad libitum-fed condition and euthanized at ZT20 (left), and corresponding densitometry analysis of relative Bmal1 protein amounts shown on the right (n=3).

(e) Densitometry analysis of relative nuclear FLAG-Bmal1 protein amounts corresponding to immunoblotting results of Fig. 3b (n=3).

(f) Densitometry analysis of relative nuclear FLAG-Bmal1 protein amounts corresponding to immunoblotting results of Fig. 3g (n=3).

(g) Immunoblot analysis of FLAG-Bmal1 and GFP (for transfection efficiency control) proteins in HEK 293T cells transfected with the plasmid expressing AdTrack-FLAG-WT-Bmal1 (1 ug) or S42A-Bmal1 (1.2 ug) and treated with cycloheximide (CHX, 10 µg ml^{-1}) for indicated times.

(h) Immunoblot analysis of FLAG-Bmal1 abundance in HEK 293T cells transfected with pcDNA, Akt or Akt2-CA, and corresponding densitometry analysis of relative FLAG-Bmal1 protein amounts shown on the right (n=3).

(i) Quantitative PCR analysis of the mRNA levels of *Dbp* in vehicle- (Ctrl) or insulin-treated (INS, 50 nM, 60 min) WT or *Bmal1* liver-specific knockout (BLKO) primary hepatocytes infected with GFP or Akt2-CA adenoviruses (n=3). Data are represented as mean ± s.e.m, statistical analyses were performed with a two-tailed unpaired Student's *t* test, *P<0.05, ** P<0.01, # no significant difference.

(j) Immunoblotting analysis of HA-tagged Clock protein amounts co-immunoprecipitated with FLAG-tagged Bmal1 wild-type or Ser42Ala mutant in the nuclear extracts from HEK 293T cells transfected with HA-Clock (0.5 µg) together with FLAG-Bmal1-WT (0.5 µg) or FLAG-Bmal1-S42A (1.5 µg).

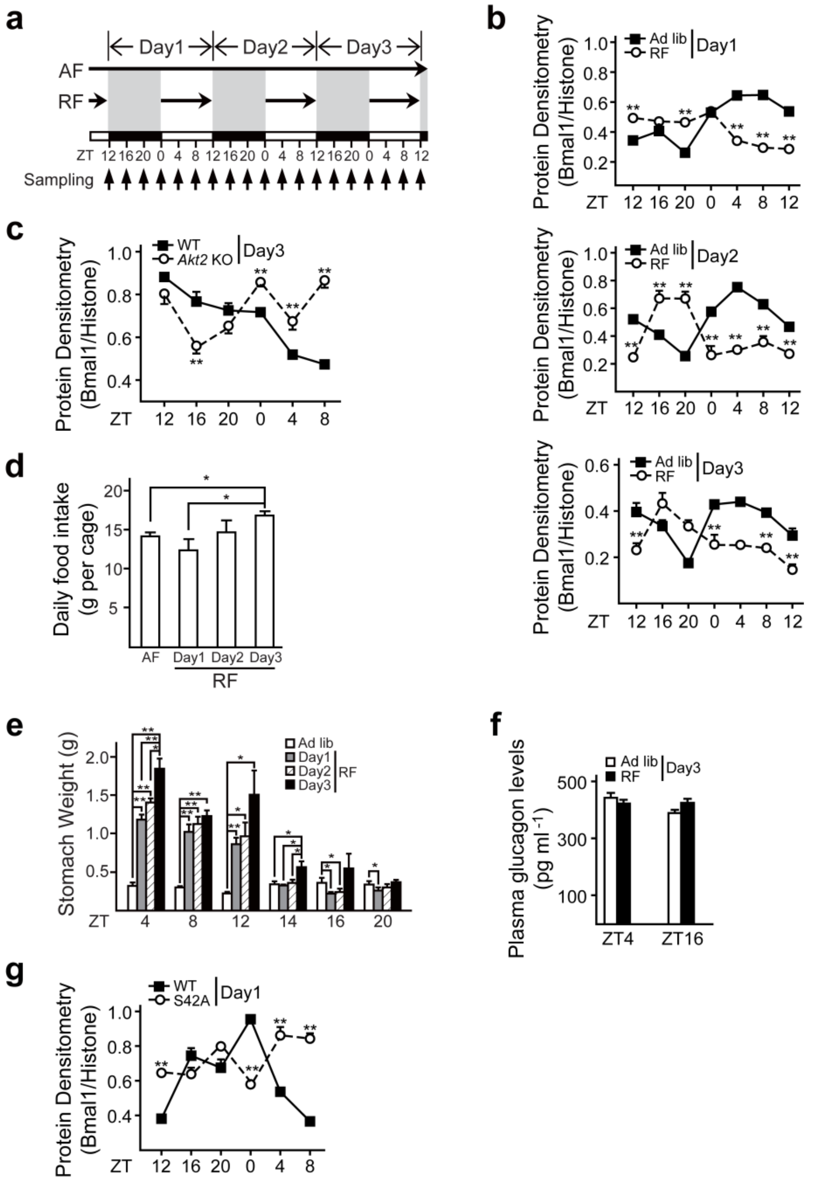

Supplementary Figure 4. Temporal effects of RF on circadian clock and appetite

(a) Experimental schedule of food entrainment. Mice were subjected to either ad libitum-feeding (AF) or daytime-restricted feeding (RF) and euthanized at time points indicated with small arrows. White bars indicate daytime. Black bars indicate nighttime. Black arrows indicate food availability.

(b) Densitometry analysis of relative nuclear Bmal1 protein amounts corresponding to immunoblotting results of Fig. 5a (n=3).

(c) Densitometry analysis of relative nuclear Bmal1 protein amounts corresponding to immunoblotting results of Fig. 5b (n=3).

(d) Measurement of daily food intake (3 mice per cage) of AF or RF mice.

(e) Measurement of the weight of mouse stomachs from AF and RF mice (n=3).

(f) Measurement of plasma glucagon levels in AF or RF mice by ELISA (n=6).

(g) Densitometry analysis of relative nuclear FLAG-Bmal1 protein amounts corresponding to immunoblotting results of Fig. 6b (n=3). Data are represented as mean ± s.e.m, statistical analyses were performed with a two-tailed unpaired Student's *t* test, * $P<0.05$, ** $P<0.01$.

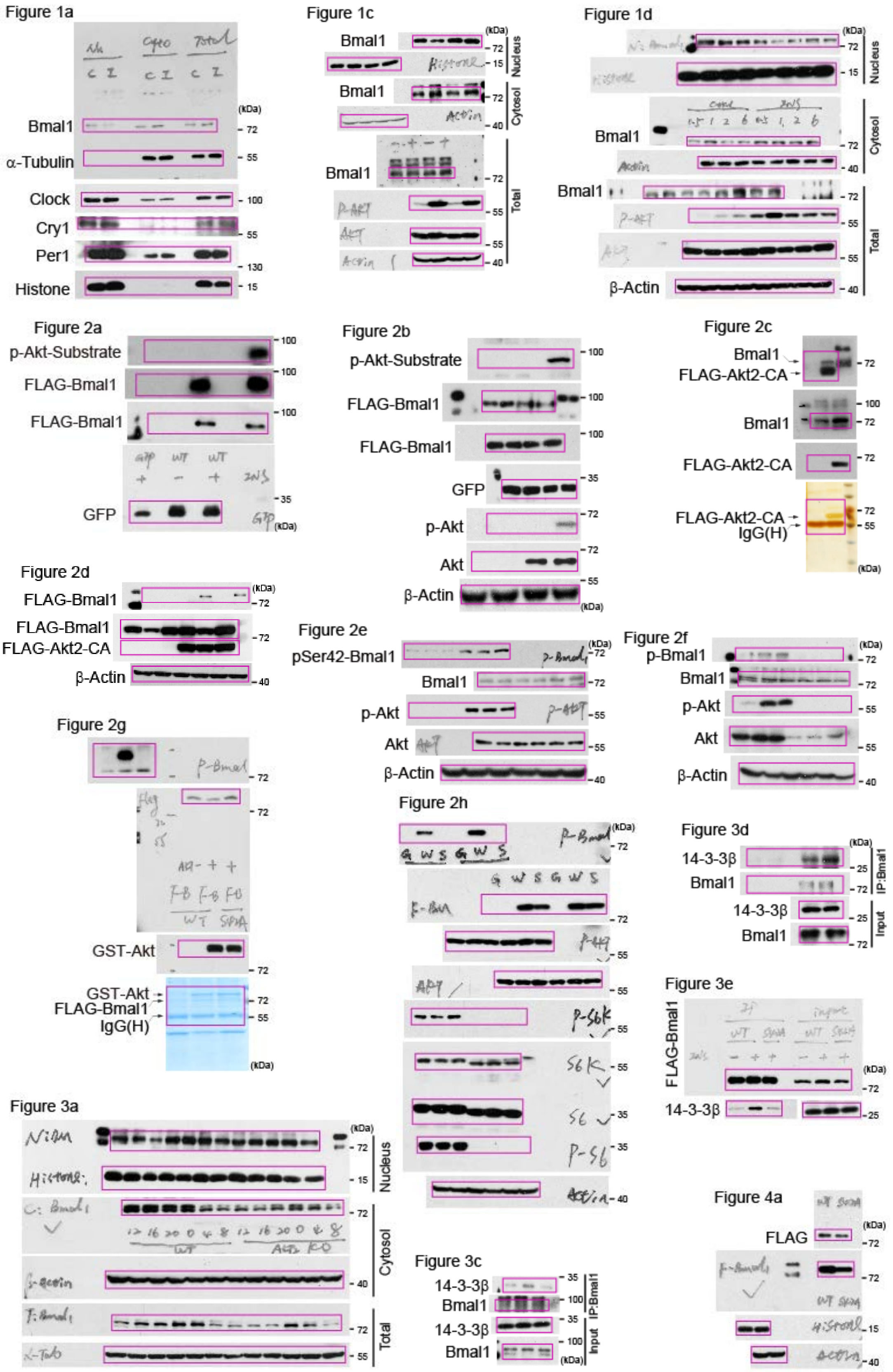
Figure 1a
Bmal1
α-Tubulin
Clock
Cry1
Per1
Histone
(kDa)
Figure 1c
Bmal1
Nucleus
Cytosol
Total
Figure 1d
Bmal1
β-Actin
Figure 2a
p-Akt-Substrate
FLAG-Bmal1
GFP
Figure 2b
p-Akt
Akt
β-Actin
Figure 2c
FLAG-Akt2-CA
IgG(H)
Figure 2d
Figure 2e
pSer42-Bmal1
Figure 2f
p-Bmal1
Figure 2g
GST-Akt
Figure 2h
Figure 3a
Figure 3c
14-3-3β
IP:Bmal1
Input
Figure 3d
Figure 3e
FLAG-Bmal1
Figure 4a
FLAG

Figure 3b

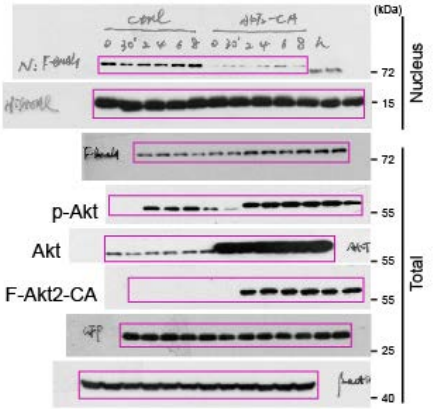

Figure 3g

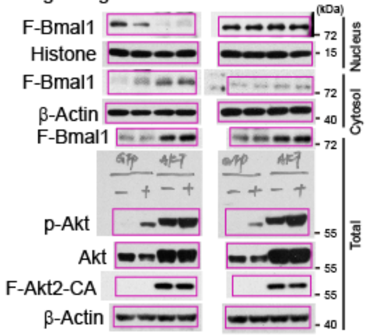

Figure 4b

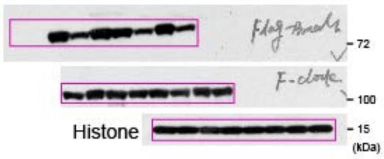

Figure 4d

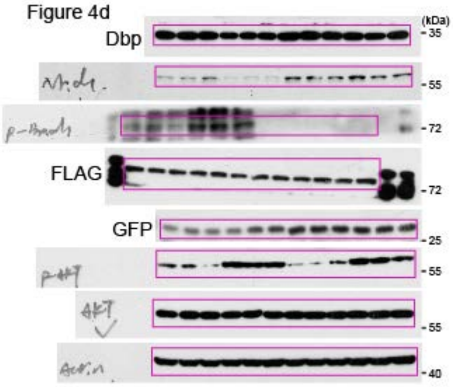

Figure 5a

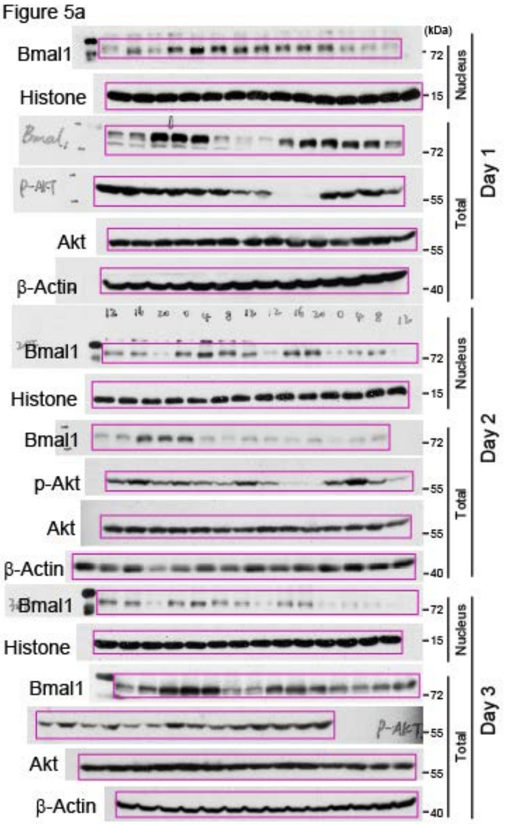

Figure 5b

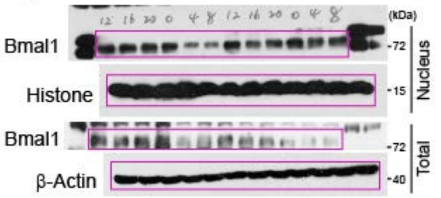

Figure 6b

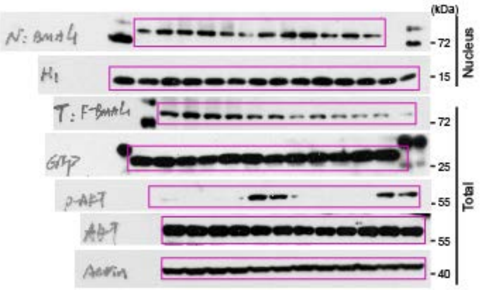

Sup. Fig. 2c

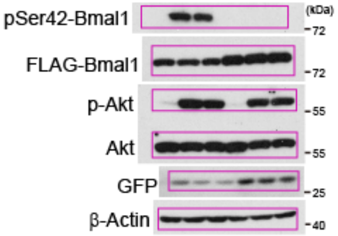

Sup. Fig. 1a

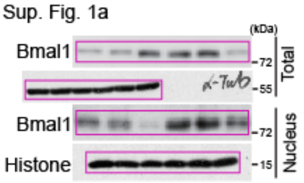

Sup. Fig. 1j

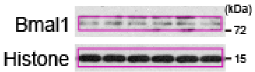

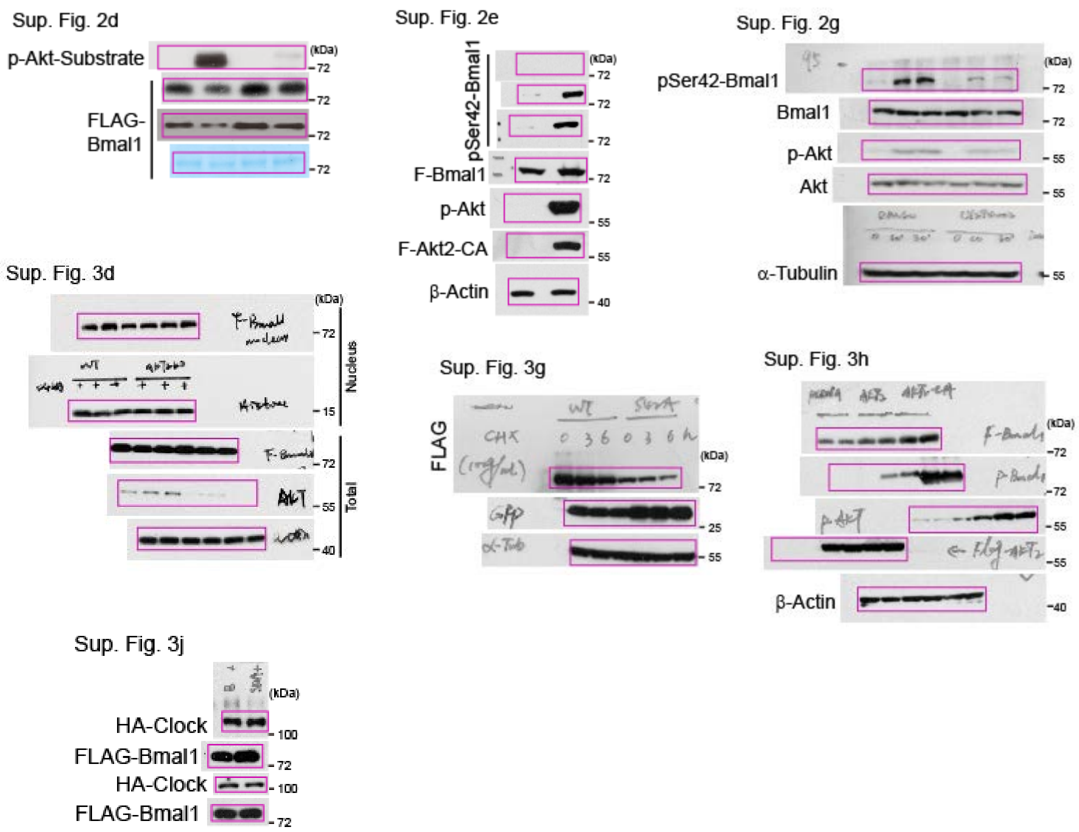

Supplementary Figure 5. Uncropped scans of all the immunoblots

Gene	Forward Primer	Reverse Primer
Bmal1	cgcggatccatggcggaccagagaatggacattt	aaggaaaaaagcggccgcctacagcggccatggcaagtc
Bmal1 (S42A)	gcaatcgcaagaggaaaggcgctgccactgact accaagaaag	ctttcttggtagtc agtggcagcgcctttcctcttgcgattgc
Bmal1 (S422A)	cgctacgaagtcgatggttcgctttc atgaacccgtggaccaa	ttggtccacgggttcatgaaagcgaaccatcgacttcgtag cg
Bmal1 (S513A)	tccacaggataagagggtcagcgccttccagctg tggctccag	ctggagccacagctggaaggcgctgaccctcttatcctgtg ga
Clock	tttggatccatggtgtttaccgtaagctg	acagaattctaactgattttttgat
Akt2	ataagaatgcggccgcgaatgaatgaggtatctgt catca	gctctagatcactctcggatgctggctgag

Supplementary Table 1. Primers used for gene cloning

Gene	Forward Primer	Reverse Primer
Bmal1	tgcagaacaccaaggaagga	attttgtcccgacgcctctt
Nr1d1	tccccaagagagagaagcaa	ctgagagaagcccaccaaag
Dbp	ctggcccgagtctttttgc	ccaggtccacgtattccacg
Per1	agcaggacaacccatctacca	cgaagtttgagctcccgaagt
Per2	ggagcaggttgagggcatta	gatggaggccacttggttagag
L32	tctggtgaagcccaagatcg	ctctgggtttccgccagtt

Supplementary Table 2. Primers used for Real-time PCR

Gene	Forward Primer	Reverse Primer
Dbp	acacccgcatccggtagc	ccacttcgggccaatgag
Nr1d1	gcttaagttcttgatttaag	tcctggggagggcctggcaag

Supplementary Table 3. Primers used for ChIP-qPCR
